# Engineered Nanomaterials in Sports and Their Environmental and Health Implications

**DOI:** 10.3390/nano16211354

**Published:** 2026-10-24

**Authors:** Huanbin Zhang, Feng Ye, Fu Jie, Saiqa Menhas, Sikandar Hayat, Xunfeng Chen, Weiping Liu, Kashif Hayat

**Affiliations:** 1 School of Physical Education, Jiangxi Tourism & Commerce Vocational College, Nanchang 330100, China; huanbinzhang1991@163.com (H.Z.);; 2 Guangdong Provincial Key Laboratory of Marine Disaster Prediction and Prevention, and Institute of Marine Sciences, Shantou University, Shantou 515063, China; 3 College of Medicine, Xi’an International University, Xi’an 710077, China; 4 Key Laboratory of Zhenjiang, Collaborative Innovation Center of Technology and Material of Water Treatment, School of Environment and Safety Engineering, School of Emergency Management, Jiangsu University, Zhenjiang 212013, China; 5 ZJP Key Laboratory of Pollution Exposure and Health Intervention, Interdisciplinary Research Academy, Zhejiang Shuren University, Hangzhou 310015, China

**Keywords:** artificial turf, athlete exposure, engineered nanomaterials, environmental fate, nanotoxicology, micro- and nano-plastics

## Abstract

Engineered nanomaterials (ENMs) are used or investigated in sports for mechanical reinforcement, friction regulation, thermal management, biosensing, energy harvesting, and tissue repair. This critical review examines their applications, environmental release, transformation, exposure pathways, biological interactions, and end-of-life management, distinguishing field measurements, laboratory tests, models, and proposed pathways. ENMs are deliberately engineered for nanoscale functionality; microplastics (MPs) and nanoplastics (NPs) are classified by polymer composition and size. Micro(nano)plastics (MNPs) are not inherently nanomaterials; nanoscale polymer particles may be engineered or incidental. MNPs from synthetic turf, polymeric infill, running tracks, footwear, and apparel are assessed as degradation products, potential carriers of embedded ENMs, and co-occurring contaminants. Field evidence is stronger for turf-derived MP release than for sports-related free ENM or NP release. Abrasion, oxidation, and weathering modify particle size, surface chemistry, and sorption capacity, while hydrological runoff, wind transport, and human activities redistribute particles. Athlete exposure through inhalation, ingestion, and dermal contact is plausible, but limited longitudinal biomonitoring warrants cautious interpretation of health risks. Laboratory ecotoxicological studies identify material- and preparation-dependent hazards to terrestrial and aquatic biota, but do not establish effects at measured sports-field drainage concentrations. Sustainability requires low-emission material design, effective physical containment, advanced drainage treatment, environmentally realistic exposure testing, transparent material disclosure, and robust end-of-life recovery systems. Circularity metrics must incorporate diffuse particulate and chemical emissions into life-cycle exposure and ecosystem risk assessments. Comparative synthesis links material properties, release identity, analytical limits, and pathway confidence; a minimum reporting framework identifies the measurements needed for quantitative exposure and risk assessment.

## 1. Introduction

Modern sports increasingly rely on highly specialized, materials-intensive environments where equipment and facilities must concurrently provide low weight, high tensile strength, rapid feedback, comfort, durability, and biological safety. Consequently, nanotechnology has been established in emerging applications across diverse sports products, including footwear, protective equipment, apparel, sensors, biomedical scaffolds, artificial turf, and court surfaces [1,2,3,4]. Nanoscale additives can enhance load transfer, stiffness, fracture resistance, electrical and thermal conductivity, wettability, antimicrobial activity, and cell–material interactions [2,3,5,6,7,8]. Alongside these performance advantages, environmental health and safety (EHS) questions remain; concern about a possible hazard does not itself establish a sports-specific dose or risk. Previous studies have emphasized the distinctive biological behaviors and EHS implications of nanomaterials (NMs), cautioning that associated risks warrant careful evaluation [9,10,11].

Sports-related engineered nanomaterials (ENMs) should be distinguished from larger polymeric materials that may release microplastics (MPs) or nanoplastics (NPs) during use and weathering. Plastics and ENMs have different production histories and material identities; widespread polymer use and deliberate nanoscale engineering should therefore be considered separately [12]. Sports-related ENMs encompass (i) metal- and metalloid-based NMs, such as titanium dioxide (TiO_2_), zinc oxide (ZnO), silver (Ag), silicon dioxide (SiO_2_), aluminum oxide (Al_2_O_3_), and iron oxide (Fe_3_O_4_); (ii) carbon-based NMs, including graphene oxide (GO), carbon nanodots (CNDs), and carbon nanotubes (CNTs); and (iii) nanocomposite materials, such as metal–organic frameworks (MOFs), nanocellulose, nanoclay, nano-hydroxyapatite, and ceramic nanostructures [2,3,5,6,7,8]. Herein, “NMs” refers broadly to materials possessing nanoscale structural features, conventionally approximately 1–100 nanometers (nm) in at least one external dimension or in relevant internal structures, with unique properties such as small size, large surface area, shape, and chemical composition; whereas “ENMs” denotes deliberately manufactured materials designed to exploit such nanoscale features. Larger polymer matrices are not NMs solely because they can fragment into smaller particles. ENMs may include engineered polymer nanoparticles; by contrast, the incidental NPs discussed here are polymer debris generated through fragmentation or wear [12]. ENMs have been the focus of much of the nanoEHS research [12]. Well-characterized ENM preparations can serve as reference materials for studying nanoscale behavior, although commercial products and their released fractions may not be uniform in size or composition [12]. In contrast, micro(nano)-plastics (MNPs) in sports environments are predominantly incidental byproducts generated through the mechanical and environmental fragmentation of larger polymeric objects, or they exist as primary additives. Conversely, the heterogeneity of most incidental NMs, including NPs, presents numerous challenges to tracking and quantifying these materials in complex environments [12]. NPs share many physicochemical properties with ENMs; however, they also present unique environmental considerations and challenges. Incidental NPs are often heterogeneous, whereas controlled ENM preparations can be more readily characterized. [12]. This analytical distinction does not mean NPs invariably pose greater exposure or risk: emission, transport, delivered dose, and material-specific effects must each be measured or modeled with stated assumptions. Consequently, NPs are a unique class of contaminants, distinct from both conventional MPs and ENMs [12], and require separate analytical and toxicological frameworks. In sports products, ENMs enable lightweight reinforcement, impact protection, sensing, ultraviolet (UV) shielding, and regenerative medicine applications. Athletes benefit from nano-reinforced materials that enhance impact absorption and equipment durability [5,13,14], while biosensors facilitate real-time physiological monitoring [15,16,17,18,19,20]. The potential for unintended biological interactions and environmental release requires rigorous lifecycle assessment. In addition, nanofiber scaffolds and nano-hydroxyapatite have been investigated for targeted tissue repair [21,22,23,24,25]. Intentionally added nanoforms, matrix-bound fillers in emitted fragments, freely liberated ENM particles or agglomerates, and dissolved ions are distinct entities. A conventional carbon fiber, bulk polymer, or device with a nanoscale feature is not by itself evidence of a freely emitted ENM.

Recent research has shown that MNPs are widely distributed across freshwater systems, soils, terrestrial biota, the atmosphere, and remote environments [26]. Here, MNPs refers to plastic particles ranging from nanometer (nm) to millimeter (mm) sizes. MNPs include primary MNPs, which are intentionally incorporated into products, and secondary MNPs, generated through the degradation of larger plastic materials [26,27,28,29]. Particle-size definitions vary: this review uses NPs for incidental polymer particles < 1 µm (1000 nm), MPs for polymer particles from 1 µm to <5 mm, and MNPs as the umbrella covering both fractions [26]. Study-specific size ranges remain attached to individual results. Material ≥ 5 mm is recorded separately where relevant to infill inventories. The NPs category adopted here therefore extends beyond the conventional 1–100 nm nanoscale and is not identical to the NMs category. Although definitions vary among studies, particle size is a critical determinant of recovery efficiency, environmental mobility, and biological interactions [12]. Terrestrial MNP sources reach receiving environments through runoff, atmospheric transport, and direct material transfer, with their relative contributions depending on the setting [30]. Policy and sustainability analyses recognize the need to reduce annual emissions of MPs by 0.5 million tons (t) [31].

**Figure 1:**
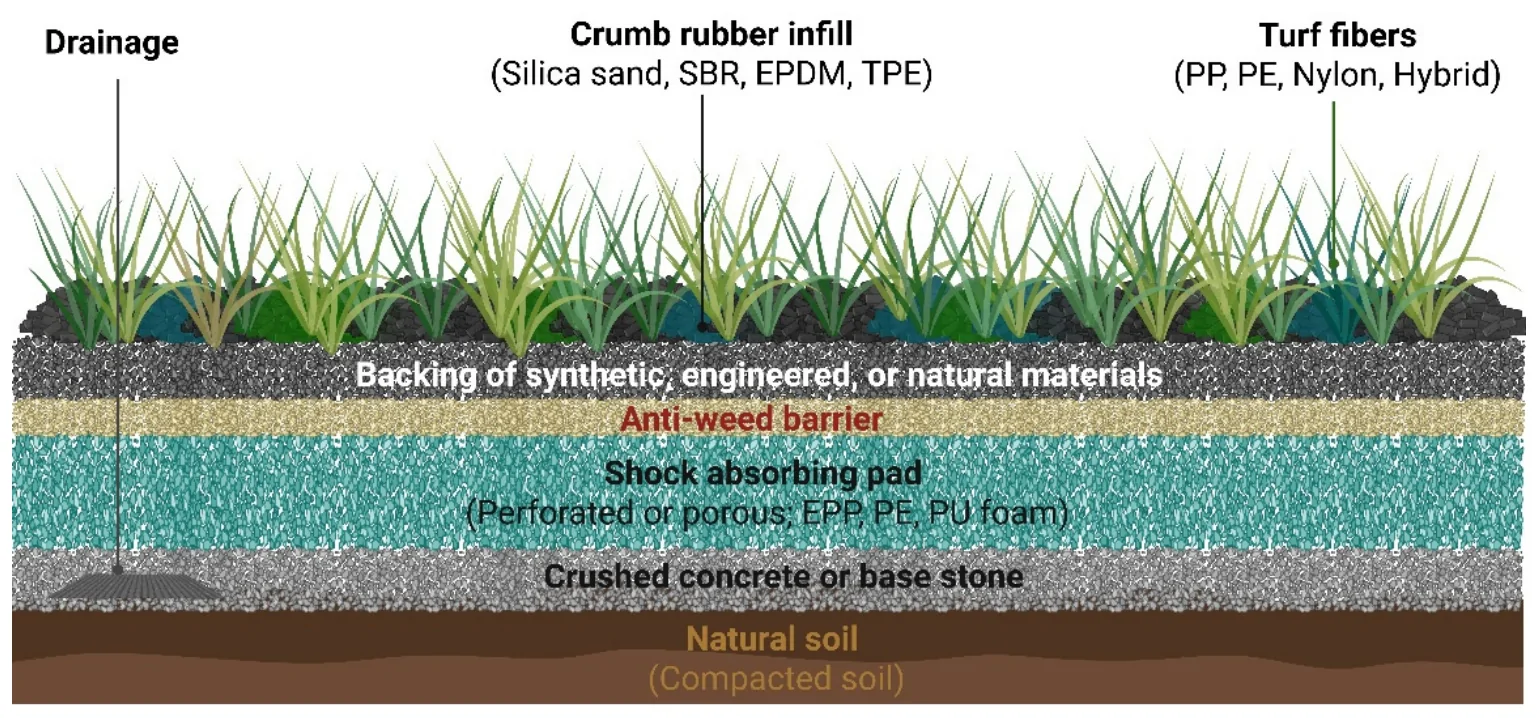
A schematic illustration of artificial turf. EPDM: Ethylene Propylene Diene Monomer; SBR: Styrene Butadiene Rubber; TPE: Thermoplastic Elastomer; PP: Polypropylene; PE: Polyethylene; PU: Polyurethane.

The same interfacial engineering strategies that improve sports products’ performance can also create pathways for releasing micro- and nanoscale particles. Mechanical abrasion, impact, coating degradation, and weathering may liberate embedded nanofillers and generate secondary MNPs and nanoparticles. For example, incorporating 0.5% nano-SiO_2_ into ethylene-vinyl acetate (EVA) shoe soles improves tensile strength; however, abrasion may produce polymer debris that contains or exposes nano-SiO_2_ [5]. Third-generation artificial turf systems consist of synthetic fibers, backing materials, shock-absorbing layers, and loose infill composed of styrene-butadiene rubber (SBR), ethylene-propylene-diene monomer (EPDM), or thermoplastic elastomers (TPEs) [27,32,33,34]. Potential released forms include intact granules, turf fibers, rubber fragments, and host-matrix particles containing fillers; freely released ENMs require separate evidence of nanofiller identity and liberation. The combined effects of mechanical stress, UV radiation, and weathering accelerate the degradation of these components, thereby producing secondary MNPs [27,32,33,34]. Consequently, the widespread adoption of artificial turf (Figure 1) has made these systems a substantial sport-related source of MP pollution [27,35]. Primary infill loss is displacement of already existing loose granules; secondary generation is abrasion or weathering that creates new smaller fragments. Neither an infill mass inventory nor a runoff MP count alone resolves their relative contribution or establishes an NP fraction.

Concerns extend beyond initial particle release to encompass source-to-receptor transport and environmental fate. Sports-derived micro- and nanoscale particles may be transported through drainage systems, wastewater, wind-driven resuspension, and anthropogenic transfer via footwear and clothing [27,36]. For example, pilot-scale production of Fe_3_O_4_-reinforced recycled thermoplastic polyurethane (TPU) and polyamide 12 (PA12) produced measured nano- and microscale aerosols across multiple production zones [37]. Particle number and size establish a process aerosol source; additional composition data are needed to attribute every detected particle to the intended nanofiller. Similarly, laboratory tests of commercial nano-enabled products reported release of 8.8–71.16% for the investigated Ti-, Zn-, or Ag-containing material under the tested conditions [36]. Such release can include particles, agglomerates, dissolved species, or material still associated with a host matrix; a total elemental-release percentage is not a percentage of freely released ENMs. However, the current literature does not establish a universal background concentration for NMs or MNPs at sports venues. Existing studies assess disparate environmental matrices and report results using different analytical units, thereby precluding the development of standardized baseline datasets for ENMs and associated MNPs.

Researchers frequently detect turf-derived MNPs in drainage systems and adjacent soils [27,28]. Their environmental fate is governed by interacting processes such as agglomeration, UV-induced oxidation, and eco-corona formation; dissolution applies to susceptible nanoforms or chemical constituents and is distinct from polymer fragmentation [36,38,39]. These processes can alter mobility and biological interactions without forming a mandatory sequence. Wang et al. [40] quantified 32,500–120,120 particles per dm^2^ on outdoor artificial turf surfaces and 8.26 × 10^6^–4.08 × 10^7^ particles L^−1^ in rainfall runoff, with >97% of particles measuring < 0.5 mm. Zhang et al. [41] examined five campus athletic fields in Beijing and reported runoff concentrations ranging from 2433 ± 493 to 5067 ± 839 particles L^−1^, with 72–81% below 1000 µm and 35–47% below 500 µm. Fibrous MPs were predominant (41–64%), followed by granules (26–45%) and fragments (8–18%). Ye et al. [42] reported mean runoff concentrations of 7.34 × 10^2^ ± 3.72 × 10^2^ particles L^−1^ from artificial lawns and 6.52 × 10^2^ ± 2.43 × 10^2^ particles L^−1^ from plastic running tracks. Direct comparisons among these values are complicated by differences in venue age, climate, sampling protocols, detection limits, and system boundaries. Current mitigation measures include hybrid turf systems and improved maintenance practices, together with ongoing research into sustainable infill alternatives [27]. Nevertheless, field-based data on emission rates remain limited, and standardized characterization methods have yet to be established. The exceptionally high [40] counts are retained as reported values with an explicit verification flag: their effective lower size limit and sampled-volume normalization have not been independently reconciled here, so they are excluded from harmonized numerical comparison and dose calculations. A reported upper-size fraction, including <0.5 mm, does not establish the lower analytical cutoff.

Although all plastics are polymer-based materials, the terms ‘plastic’ and ‘polymer’ are not interchangeable, nor are ‘NPs’ and ‘nanopolymers’ synonymous [12]. By definition, plastics consist of polymeric matrices combined with various additives, whereas polymers alone refer strictly to the macromolecular constituents. NMs and polymers provide biomechanical benefits and are investigated for biomedical applications [5,13,14,21,22,23,24,25]. Experimental evidence indicates that some NMs can induce pulmonary, hepatic, or systemic inflammation [1,39]. Elevated ventilation rates during strenuous physical activity may increase inhaled particle doses, but any resulting increase in athlete health risk remains to be established. Furthermore, self-powered sensors generate polymeric and conductive NM waste streams [17], while nanomedicine applications require controlled material degradation to prevent adverse biodistribution [43]. Occupational exposure risks also affect facility workers and manufacturing personnel [37]. MNPs and fibers generated through environmental degradation have emerged as important contaminants within the food chain [28]. Their toxicological profiles are determined by particle size, surface chemistry, solubility, and additive composition [1,39,44]. Mechanistic studies indicate that MNPs can induce oxidative stress, disrupt epithelial barriers, and dysregulate the immune system. However, translating these hazards into sports-specific risks requires realistic exposure data [28,29,33]. Relevant exposure pathways include the inhalation of resuspended dust, incidental ingestion, and dermal contact with abraded skin or infill-contaminated clothing [27,44]. Each exposure route produces distinct biodistribution patterns influenced by the physicochemical properties of the particles [1,39,44]. Despite exercise-induced physiological alterations, athlete-specific personal sampling data and longitudinal epidemiological evidence remain scarce.

Ecological studies provide material-specific observations along the source-to-receptor chain, but measured occurrence, laboratory response, and field effects remain separate evidence. However, ecotoxicological studies identify potential hazards, although their relevance to sports-field exposures requires further evaluation. EPDM infill can accumulate locally; substrate experiments across 0–64% infill by volume and a separate 5% by-volume competition treatment demonstrate loading-dependent effects on grassland species, including *Plantago lanceolata* (*P. lanceolata*) [45]. These experimental treatments do not define a universal 5% field toxicity threshold. Aquatic studies document ingestion of artificial-turf fibers and toxicity of mechanically generated EPDM-NPs from granule surfaces to *Daphnia magna* (*D. magna*; water flea), with no significant toxicity reported for particles derived from the granule core [46,47,48]. Effective mitigation, therefore, requires lifecycle-based controls rather than material substitution alone. Potential strategies include low-shedding fibers, strongly bound nano-coatings, reduced loose infill, perimeter containment systems, drainage filtration, and extended producer responsibility (EPR). However, each intervention entails trade-offs involving performance, cost, and climatic suitability [27,35,49]. Recycling also introduces a “circularity paradox,” whereby downcycling polymers reduces demand for virgin resources but may increase diffuse MP emissions in secondary applications [50,51]. The EPDM abrasion suspension is not the measured drainage-water concentration; the preparation, organism, and chemical fraction must be matched before an ecological exposure–response inference is made.

Despite the growing use of nanotechnology in sports [27,36,37,39,44], critical knowledge gaps remain. Terminology and analytical methods are not harmonized across particle-size scales [27,36,39], limiting meaningful inter-study comparisons. Real-world emission rates, matched background datasets, and source-apportionment data remain poorly quantified [2,5,27,36,38,52]. Athlete exposure data and integrated dosimetry models that account for exercise-induced physiological changes are also scarce [21,27,36,37,39,43,44]. In addition, mixture-toxicity studies rarely employ field-aged particles, while the geographic distribution of research is heavily skewed toward Europe and parts of Asia [27,34,35,49,51]. Studies of nanoscaffolds and hydrogels predominantly evaluate short-term biocompatibility, with limited attention to long-term degradation, migration, and clearance [21,22,23,24,25]. Finally, few studies assess performance and safety concurrently, leaving the lifecycle benefits and circularity of nano-enabled sports products largely unresolved [2,5,8,16,37,53,54].

Sports-specific evidence requires an explicit sports product, surface, venue, sports-relevant test configuration, or athlete population, together with a measured property, release, occurrence, exposure, biological endpoint, or stated model. Generic composites, laundering, skin studies, tire chemistry, and toxicology are included as mechanistic or contextual evidence and identified accordingly. Intentional therapeutic administration and contained anti-doping use are retained to explain their distinct exposure and disposal boundaries. Material-performance studies are not counted as field release studies, and a nano-enabled product is not assumed to be commercially prevalent. Evidence is appraised separately for source identity and sports specificity; sampled matrix and setting; size window and chemical identification; release form and dissolved fraction; dose and duration; endpoint and controls; and model assumptions. Field observations, laboratory tests, models, and hypotheses retain these labels. Conflicting counts are compared only after aligning matrix, denominator, effective size recovery, event conditions, and system boundary. Differences that remain unresolved are reported as uncertainty rather than averaged. Earlier sports-nanotechnology reviews [9,10,11] primarily organize material applications and performance, while turf-focused reviews [27,34,35] examine synthetic-surface emissions and environmental concerns. The present contribution aims to connect these domains without substituting MP occurrence for free-ENM release or laboratory hazard for measured field risk. The lifecycle comparison additionally distinguishes resource savings from diffuse particulate and chemical loss. Accordingly, this review focuses on ENMs in sports products, integrating performance engineering with a source-to-pathway-to-receptor framework from product design and release to exposure, mitigation, and end-of-life management. MNPs are addressed as potential carriers of embedded ENMs, secondary degradation products, and co-occurring contaminants relevant to release assessment; evidence for polymer particles is explicitly distinguished from that for free ENMs. Specifically, our review aims to distinguish demonstrated benefits from reported hazards, critically evaluate the robustness of current conclusions regarding athlete risk, and establish a research and governance agenda that sustains sports performance while minimizing adverse effects on ecological and human health.

## 2. Sources, Distribution, and Occurrence of ENMs in Sports Products and Related MNP Releases

Nanotechnology is used or investigated in athletic apparel, equipment, and infrastructure [11]. The following examples distinguish sports-oriented prototypes from contextual material tests. Footwear represents a substantial reservoir of nano-enabled materials, as soles, midsoles, and uppers undergo millions of loading cycles. Nanofillers, including nano-SiO_2_, nano-calcium carbonate (CaCO_3_), GO, CNTs, and conductive nanoparticles, are incorporated into polymers such as EVA, TPU, and thermoplastic rubber (TPR) to enhance structural stability and load resistance [5,14]. For example, GO-reinforced soles may improve load distribution, suggesting an injury-prevention hypothesis that requires validation of clinical outcomes [14]. Shear-thickening fluids containing nano-SiO_2_ or CNTs exhibit pronounced increases in viscosity and energy absorption under high-strain impacts [55,56], whereas conductive nanofillers impart quasi-piezoelectric properties that enable impact sensing [57]. These mechanical and sensing results establish functionality under testing. Abrasion of a nano-enabled sole is a proposed source of polymer-matrix debris, while a sports-field emission factor for freely released nano-SiO_2_, GO, or CNTs remains unresolved.

Nanocomposites are used in helmets, rackets, and bicycles, where carbon-fiber reinforcement and nanoscale additives such as graphene and nanoclays can enhance stiffness and durability [6,58,59,60,61,62,63,64,65]. However, this diverse equipment portfolio also constitutes a potential source of particulate release. Evidence suggests that matrix incorporation does not guarantee permanent immobilization, as mechanical fatigue, UV weathering, and end-of-life processing can release host-matrix fragments or free NMs [66]. In protective equipment, NMs enhance energy dissipation, whereas nanocoatings provide waterproofing and optoelectronic functionality [67]. For example, nickel (Ni)-cobalt (Co)-SiC coatings prepared for a leg-press-machine rod show improved microhardness and wear resistance under the reported electrodeposition conditions [13], while UiO-66-NH_2_@DOPO-modified MOF functions as a nanoscale flame retardant, increasing the limiting oxygen index to 31.2% and reducing total smoke production by 80.64% [68]. Additive manufacturing further broadens the range of potential sources by incorporating multi-walled CNTs (MWCNTs) into customized padding [69]. MWCNTs have also enhanced the impact resistance of basalt-fiber laminates [70]. The foam and laminate results describe laboratory properties, rather than measured releases from helmets, gym equipment, or padding during play. Conventional carbon fibers should not be counted as ENMs without documented nanoscale dimensions.

At the athlete–equipment interface, NMs regulate force and energy transfer. Graphene nanoparticles reinforce aluminum (Al) alloys used in table-tennis equipment, whereas soccer-ball nanocomposites optimize carbon–epoxy interfacial interactions [63,64,65]. Researchers have evaluated metal-matrix nanocomposites, such as carbon-reinforced magnesium (Mg) alloys manufactured using ultrasonic cavitation, for their strength-to-weight ratios [7,71,72,73,74,75,76,77]. For instance, a ZK60 Mg-alloy hybrid containing 3% short carbon fibers and 5% nano-B4C achieved a tensile strength of 348 MPa and an energy absorption capacity of 38.4 J/cm^2^, suggesting potential for bicycle-frame applications [72]. Fiber–polymer systems similarly dominate the manufacture of lightweight sports equipment. Incorporating nano-ZnO, nano-SiO_2_, or graphene into natural- and synthetic-fiber laminates improves their mechanical properties [78,79,80,81,82]. GO-reinforced Kevlar/banana-fiber laminates and nanocellulose composites have further broadened the use of renewable feedstocks in sports materials [83,84,85]. A piassava/Kevlar composite containing 1.5 wt.% SiO_2_ nanoparticles achieved tensile and flexural strengths of 336 and 381 MPa, respectively [79]. In graphene-filled natural-fiber epoxy composites, optimal flexural and tensile properties occurred at 6 wt.% graphene, whereas 8 wt.% yielded the highest hardness and lowest water absorption [86]. Electrospun fiber nanocomposites provide an additional strategy for nanoscale reinforcement [87]. However, optimal performance is generally confined to narrow nanoparticle-loading ranges, as excessive concentrations promote agglomeration and weaken interfacial bonding, which may create preferential failure sites; direct release measurements need to be established [70,88,89,90,91]. For example, hexagonal boron nitride (hBN) has improved the compatibility between carbon fibers and PA12 [90], whereas titanium diboride (TiB_2_) nanoparticles have increased the flexural strength, stiffness, and impact toughness of aramid sandwich composites by 27.6%, 62.5%, and 104%, respectively [89]. Interfacial engineering may therefore help limit structural failure, but a reduction in environmental release must be tested rather than inferred from strength alone. Nevertheless, field-derived emission factors for representative nano-enabled equipment under actual athletic conditions remain unavailable [66]. Advanced manufacturing techniques, including automated fiber placement and topology optimization, enable the production of complex geometries, but also generate particulate waste [2]. Hybrid nanofillers have been extensively reviewed as modifiers to enhance impact performance [88]. In addition, nano-modified polyacrylonitrile (PAN) fibers have been shown to improve carbon-fiber conversion and the resulting material properties [92], while nano-alumina fiber–metal laminates have provided high specific strength and improved interlaminar behavior [93]. Machining carbon-fiber-reinforced polymers (CFRP) and hybrid nanocomposites induces delamination and releases micro- and nanoscale debris [2,94]. Furthermore, polymer metallization and the incorporation of nanocellulose into polyester resins create multilayered structures that complicate end-of-life recycling [95,96,97,98]. Friction-stir-processed AZ91D/AgNP/TiO_2_ hybrids have demonstrated that nanoparticle fraction and processing conditions jointly influence tensile performance [71]. The alloy, natural-fiber, sandwich, and machining studies remain as contextual evidence where they do not test a sports venue or athlete. Table 1 compares the reported property changes within individual test boundaries; however, it does not rank environmental safety across different formulations.

Sports facilities constitute extensive, long-service-life reservoirs of nano-enabled materials [100]. CNT-reinforced PVC flooring, nanocomposite athletic tracks, and nano-SiO_2_-modified concrete provide enhanced durability, hydrophobicity, and self-cleaning properties [100,101,102,103,104]. Graphene- and CNT-reinforced panels have also been modeled for stadium roofs to improve structural damping [105,106,107,108,109,110,111]. Although these infrastructural innovations may reduce maintenance frequency, NMs may be released episodically during extreme weathering, resurfacing, or demolition. Lifecycle assessments must, therefore, account for deconstruction, a critical driver of composite dust generation. Structural calculations and prototype durability results do not establish free-ENM occurrence in venue air, runoff, or demolition dust. The material composition and identity of each emitted fraction must be verified at those lifecycle stages.

Artificial turf and winter-sport surfaces illustrate how performance interfaces can become direct environmental sources of MNPs [27,112]. Artificial-turf systems consist of synthetic fibers, backing materials, shock pads, and performance infill (Figure 1), including SBR, EPDM, TPEs, or cork [32,33]. In the United States alone, tens of millions of tires have been used in artificial turf fields and as playground surface coverings [33]. A single professional field requires approximately 20,000–40,000 tires’ worth of crumb rubber, with continued replenishment throughout its operational lifespan [33]. Crumb rubber is chemically complex, containing natural and synthetic rubbers, vulcanization chemicals, and antioxidants. Consequently, particles within the MP size range can function as chemical reservoirs and sorbents. Mechanical stress and weathering can release MNPs and expose or mobilize associated chemicals, including lead (Pb), Zn, and persistent organic pollutants (POPs); particles, dissolved ions, and organic leachates are separate measurement targets [27,28,46,47,48]. The principal mechanisms of MNP release include the direct displacement of loose infill, abrasion in high-friction zones, photochemical aging, and fragmentation during maintenance [27,34]. Rubberized tracks and court coatings further contribute polyurethane and organosilicon particles. Wang, Sun, Zheng, Xiang, Xu, Xiang and Fan [40] reported that artificial turf contained the highest surface abundance of MNPs compared with plastic tracks and hard courts. Unlike discrete sports equipment, artificial turf’s extensive surface area and prolonged service life result in emissions governed by complex interactions among material aging, climatic conditions, and drainage dynamics. They report primary granule migration from play, runoff, footwear transfer, maintenance, or snow handling separately from secondary abrasion of fibers, granules, backing, and track coatings. Available inventory losses and selected runoff observations do not support a transferable percentage contribution for the two mechanisms. MNP release from artificial turf comprises two distinct pathways: primary infill loss and secondary particle generation. Current field evidence robustly identifies primary infill loss as the dominant near-term mass-loss pathway because it is directly quantifiable through refill accounting, runoff monitoring, and maintenance records. Conversely, while secondary particle generation is mechanistically plausible and supported by morphological evidence of weathering (e.g., surface cracking and fragmentation of synthetic fibers and shock pads), it remains poorly quantified in situ. The literature currently lacks standardized measurements of degradation rates and direct emission fluxes, precluding robust estimates of annual secondary emission rates. Consequently, although both pathways are recognized, existing studies cannot yet definitively partition their relative contributions to total MNP emissions, a balance that remains highly contingent upon field design, age, and maintenance protocols.

Winter sports employ specialized nanocoatings to reduce surface drag and nanolubricants to minimize friction in machinery [52,112,113]. High-shear operation and contact with meltwater are potential mobilization mechanisms; their sports-specific emission magnitudes remain product- and setting-dependent. Mass-loss estimates illustrate the magnitude and uncertainty of these emissions. van Kleunen, Brumer, Gutbrod and Zhang [45] reported annual infill losses of 1.5–2.5 t per field, rather than measuring those losses in their plant experiment, whereas the dynamic material-flow model of Sundan, Michelsen, Rasmussen, Aas and Bohne [51] estimated that 1187 t of SBR were lost in Norway in 2020, corresponding to approximately 903 kg per field annually. At the European scale, the scoping review of Bø, Bohne and Lohne [35] summarized an estimate of artificial-turf MP release of approximately 16,000 t of MPs annually. These estimates should be interpreted as emission indicators rather than as measurements of concentrations in receiving environments. Within individual fields, high-traffic zones, drainage sumps, and maintenance depots function as accumulation hotspots (Table 2). Consequently, a grab sample reflects the event it captures and may not represent an annual load, a hotspot maximum, or a receptor dose.

Sports textiles and wearables represent critical interfaces for direct exposure of both athletes and the environment. NMs have been incorporated into sportswear to provide water repellency, antimicrobial activity, and UV protection [54,114,115]. Beigzadeh et al. [116] reviewed nano-textile release during use and washing, with reported released fractions ranging from less than 1% to nearly complete loss across different products and protocols. Ag release from functionalized textiles reached up to 45%, depending on the binding chemistry and degree of abrasion. Likewise, TiO_2_-functionalized cotton exhibited 70% water repellency and 99% bacterial elimination [54], whereas copper (Cu) nanoparticles enhanced the color, UV-protective, and antimicrobial properties of polyester [115]. The shift toward “smart” nanotextiles has driven the integration of piezoelectric nanofibers and conductive hydrogels for biomechanical sensing and energy harvesting [4,16,38,117,118,119,120,121,122,123]. However, discarded wearable devices contribute to electronic (E-waste) and nano-enabled material waste [11,124]. Although nanocellulose and natural nanofibers are promoted as sustainable alternatives, inhalation hazards depend on the emitted form and aerosol size, and their composite matrices often contain persistent synthetic resins [3,8]. Nanoparticles’ proximity to the skin, combined with their release into wash water, increases the potential for dermal exposure and aquatic contamination. Nano-enabled topical products further extend these exposure pathways into outdoor environments. Lehutso et al. [125] characterized 18 commercial nano-enabled products spanning sunscreens, personal care, clothing, and paints. Seventeen of these products contained ENMs, including nano-TiO_2_ (nTiO_2_), nAg, and mixed nZnO/nTiO_2_ or nTiO_2_/nAg systems, with particle dimensions ranging from tens to several hundred nm. Such product inventories identify materials requiring release tests for sweat contact, showers, swimming pools, clothing, and runoff; product content alone is not a measured source term for these matrices. However, they do not establish environmental concentrations within sports facilities, indicating the need for direct monitoring at these venues.

Mechanical friction, exposure to sweat, and repeated laundering should be included in assessments of flex-fatigue resistance and leachate toxicity; the device-performance studies [126,127,128,129,130,131] do not by themselves quantify nanoparticle detachment from garments. Conductive hydrogels have demonstrated high stretchability, crack resistance, water stability, antifreezing properties, and ionic conductivity [128]. For example, a degradable polyvinyl alcohol (PVA)-gelatin elastomer monitored joint angles with a 75 ms response time and classified foot-arch and walking states with accuracies of 97.5% and 99.6%, respectively [129]. A PVA/O-carboxymethyl chitosan organohydrogel containing Ag nanoparticles achieved a conductivity of 1.4 S/m and a fracture stress of 2.2 MPa for sports-monitoring applications [127]. Similarly, a chestnut-tannin/Ag hydrogel operated at −20 °C exhibited a fracture strain of 350.54% and a tensile strength of 1.55 MPa, and measured velocity with an error of less than 3% [126]. Response time, classification accuracy, conductivity, fracture strain, and low-temperature operation are performance endpoints that should be combined with encapsulation-integrity and entity-resolved release measurements before claiming low-emission use.

**Table 2:** Sports-specific quantitative observations of polymeric materials.

Study and Setting	Quantity and Matrix	Size Boundary	Identification or Estimation	Principal Uncertainty	Reference
Field occurrence (outdoor synthetic surfaces)	Surface MPs: 32,500–120,120 items dm^−2^; MPs in rainfall runoff: 8.26 × 10^6^–4.08 × 10^7^ items L^−1^; >97% reported < 0.5 mm	<0.5 mm	Not available (NA)	Unreconciled lower size limit, identity confirmation, and count/volume normalization; excluded from pooled comparison, dose and risk calculation.	[40]
Athletic field runoff (five campus field types in Beijing)	MPs in stormwater runoff: 2433 ± 493 to 5067 ± 839 particles L^−1^. Reported fractions: 72–81% < 1000 µm; 35–47% < 500 µm	0–5000 μm	ATR-FTIR and µ-FTIR identify EPDM, SBR, PE, PP, and other reported fibers.	The results are derived from specific experimental conditions and samples. Single-campus coverage; confirmation window and inclusion of rayon affect comparison.	[41]
Field runoff (Six venues in Chongqing)	Average MP abundance in runoff from artificial lawns = 7.34 × 10^2^ ± 3.72 × 10^2^ particles L^−1^; plastic running tracks = 6.52 × 10^2^ ± 2.43 × 10^2^ particles L^−1^. Eighteen wet-season samples. >90% of total MPs reported were <0.5 mm	<0.5–5 mm	Microscopy with stratified µ-FTIR confirmation; reported counts corrected by 91.3% confirmation rate	Preliminary evidence; limited events and venues; partial confirmation and urban background; no NP measurement.	[42]
Greenhouse compartments	5% (corresponding to c. 22,000 particles per kg of substrate)	Infill material: 0.5–2.5 mm EPDM and 0.5–2.0 mm cork	Root scanning; linear mixed and binomial generalized linear mixed models	Greenhouse study; losses do not quantify receiving-water concentration or new fragments.	[45]
Artificial football turfs	1187 t of SBR lost to the environment; ~903 kg per annually operated field	NA	Dynamic material-flow analysis	Field stock, loss assumptions, management, and system boundaries; not free-ENM or NP emission.	[51]
Field drainage (synthetic football field); Laboratory abrasion EPDM granules in water	Trace EPDM-related NPs; no polymer-specific ambient NP count used for risk calculation. EPDM-related NPs = 4.3 × 10^8^ ± 5.8 × 10^7^ particles mL^−1^	<50–233 nm	Concentration/fractionation and attenuated total reflectance Fourier-transform Infrared spectroscopy (ATR-FTIR); Nanoparticle tracking analyses (NTA) characterizes detectable particles. ATR-FTIR, NTA, and transmission electronic microscopy (TEM) characterize generated preparation.	Hazard suspension, not field drainage concentration; particle/chemical causation unresolved.	[48]
Material chemical survey and modeling of turf infill	1.60–5089 ng g^−1^ total volatile methylsiloxanes in synthetic turf infill	NA	GC-MS analysis; targeted chemical analysis and modeled oral/dermal exposure	Chemical-specific; no nanoparticle counting, inhalation assessment, or full-mixture safety.	[132]

The boundary between sports equipment and health technology is becoming increasingly indistinct. Nano-enabled biosensors incorporating nano-Al_2_O_3_, graphene, or nano-gold electrodes can monitor lactate and electrolyte concentrations in sweat and saliva [18,19,20,133,134,135,136,137,138]. Nanostructured analytical platforms have also been investigated for supplement analysis and anti-doping detection [139,140,141,142,143], whereas nanomedicine concerns intentional internal delivery in the treatment of sports-related injuries. Nano-liposomes improve nutrient bioavailability, whereas nanocomposite scaffolds made from materials such as nano-hydroxyapatite, graphene, and silk fibroin facilitate tissue regeneration after fractures and tendon ruptures [21,22,23,24,25,43,144,145,146,147,148,149,150,151,152,153]. These applications exploit the high specific surface area and controlled degradation properties of NMs, including ENMs. Formulation and exercise-related physiology can be relevant to carrier behavior, but the investigations [154,155,156] do not provide a general sports-specific exposure or biodistribution model. Green synthesis of ZnO nanoparticles has been proposed for sports rehabilitation [157], while micro-nano scaffolds have been evaluated for treating dance-related leg injuries [158]. Exercise also affects inflammation, oxidative stress, and the physiological environment in which nano-encapsulated nutrients function [154]. These applications create a dual material flow: diagnostic wearables generate chemically complex, short-lived solid waste requiring appropriate containment, whereas nanotherapeutics produce controlled but potentially persistent internal exposure. Although these technologies show therapeutic potential, comprehensive safety assessments that address long-term biodistribution and degradation kinetics remain indispensable to mitigate risks at this intimate athlete-NM/ENMs interface. These clinical and analytical examples are contained within distinct boundaries: therapeutic administration, controlled degradation, device containment, and laboratory disposal. They are not evidence that athletes inhale engineered particles emitted from equipment during routine play.

## 3. Environmental Fate of ENMs and Associated Polymer Particles in Sports

**Figure 2:**
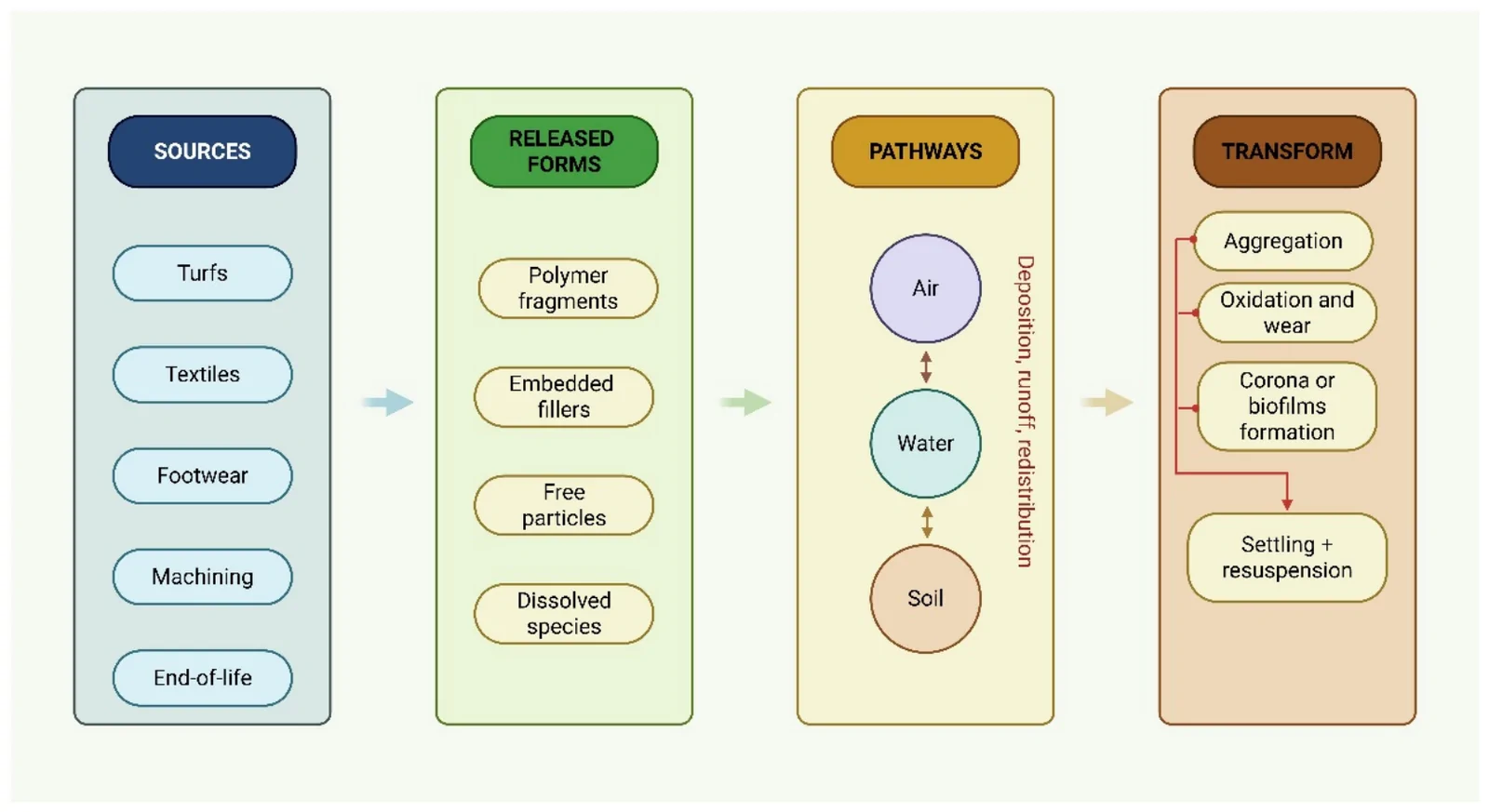
Release, environmental pathways, and transformation. Sports-derived mixtures move through air, water, and soil, while agglomeration, dissolution, oxidation, biofilm formation, settling, and remobilization alter fate.

The environmental trajectory of NMs and MNPs encompasses their generation, transformation, transport, and subsequent biological interactions. Material architecture and operational stresses govern their release (Figure 2). Surface-bound nanoparticles may detach directly, whereas embedded particles generally require degradation of the surrounding matrix. Release forms thus occur along a continuum; for instance, an embedded ENM may remain encapsulated within a polymer fragment, become exposed on a weathered surface, detach as an agglomerate, or partially dissolve. Similarly, a mm-scale turf granule can migrate intact before abrasion produces smaller MPs or NPs; a granule at or above 5 mm is an inventory item rather than an MP under the convention adopted here [66]. Consequently, the pristine NMs used in manufacturing may differ substantially from the secondary micro- and nanoscale particles encountered in the environment. Table 3 records the released entity, matrix actually studied, analytical identification boundary, likely receptor, and evidence type. A proposed receptor pathway is labeled as such when contact or uptake has not been measured.

Mechanical degradation, including abrasion, impact, and fatigue, along with physicochemical weathering such as oxidation and hydrolysis, drives these emissions. Mechanical abrasion is particularly intense in sports environments because of repetitive foot strikes, cleat-fiber contact, and equipment impacts [27]. UV exposure and oxidation can embrittle polymers, altering both the quantity and size distribution of released particles. Therefore, emissions from aging artificial turf may exhibit nonlinear temporal patterns [27,34]. Accurate source apportionment, therefore, requires comprehensive characterization of the product matrix, particle morphology, and ionic speciation. Source-attributed field measurements currently support the occurrence of polymer particles on selected synthetic surfaces. Generic abrasion tests establish possible release mechanisms; they cannot replace liberated-ENM measurements from a specific shoe, racket, helmet, or venue.

Laundering strongly influences the release of NMs and/or ENMs from textiles by combining mechanical deformation with exposure to surfactants. This process detaches microfibers and surface-associated particles, while variations in ionic strength regulate particle agglomeration [36]. Particle behavior in deionized water therefore does not adequately represent what occurs in sweat or natural aquatic systems. Released Ag may occur as discrete particles or dissolved species, whereas TiO_2_ and ZnO may remain matrix-bound, disperse as particles, or acquire eco-coronas; dissolution and speciation depend on composition and medium. Accordingly, total elemental concentrations are inadequate proxies for ENM exposure unless accompanied by characterization of particle size and chemical speciation [125]. The future development of “smart” textiles should integrate emission profiling across repeated wash cycles, size-resolved quantification of released particles, and assessments of retained functionality [3,4,38,54,115].

**Figure 3:**
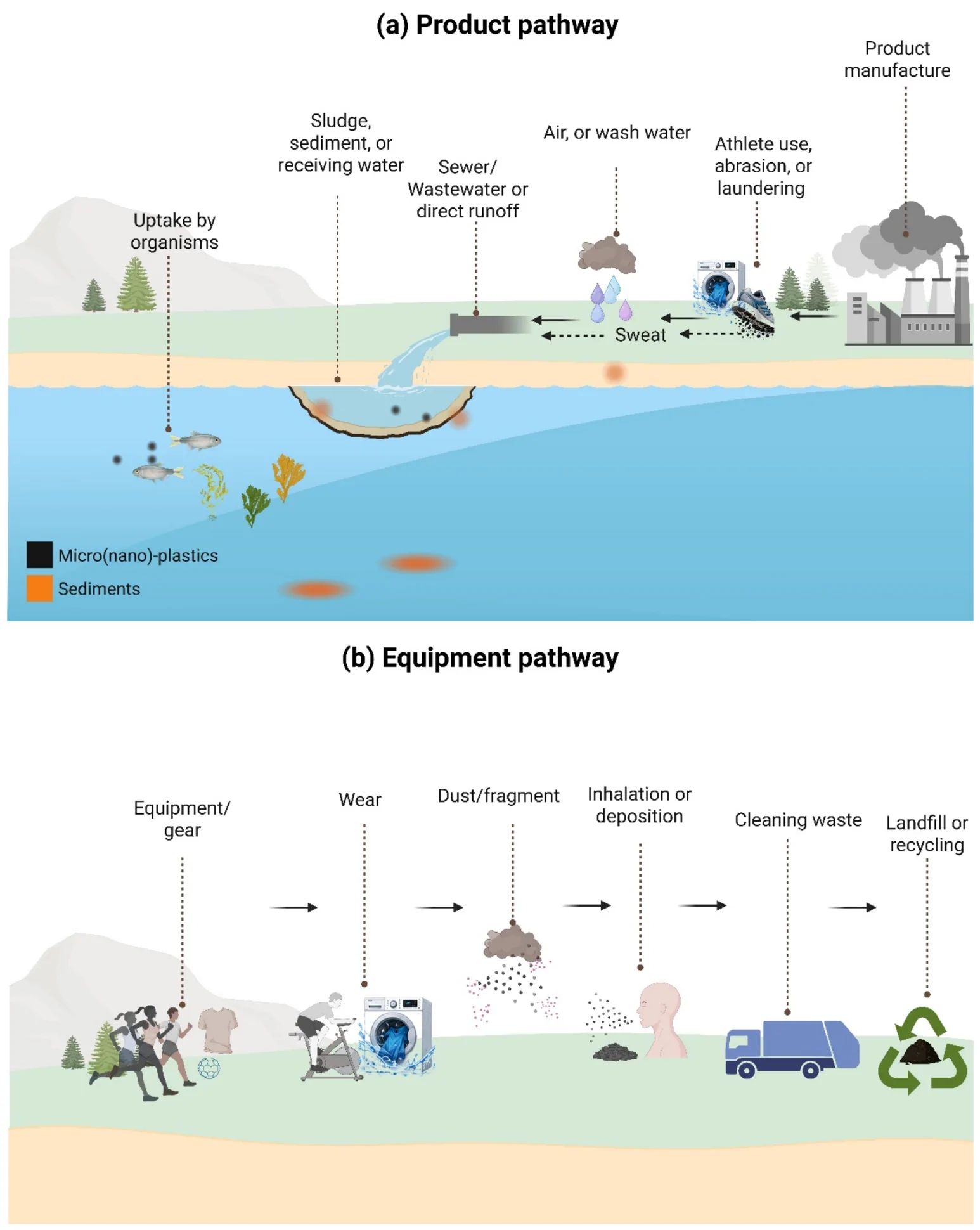
Lifecycle of products in the sports industry. (**a**) Product pathway; and (**b**) equipment pathway.

A practical sports-product lifecycle involves two primary pathways (Figure 3). Footwear and protective composites incorporate nanofillers, such as nano-SiO_2_, to improve wear resistance [5,55,56,89]. Mechanical degradation during athletic activity may release these particles; however, associated emissions remain inadequately quantified. Standardized wear-testing protocols that simulate running and cutting dynamics are therefore needed to quantify particle release in terms of both number and mass and to distinguish matrix-bound particles from liberated fractions.

Manufacturing processes can generate short-duration exposures to nano- and microscale aerosols. Pilot-scale studies have quantified nano- and microscale emissions during the compounding and extrusion of NM-reinforced polymers used in 3D printing [37]. Similarly, machining carbon fiber reinforced polymers (CFRPs) generates particulate waste [94], while composite recycling and shredding require separate dust and constituent-release assessment [53]. These occupational hazards affect workers involved in both production and recycling. Although occupational controls can be implemented immediately [37,94], field studies are required to determine whether athlete exposure is primarily driven by freshly generated micro- and nanoscale particles or resuspended dust. Removal and resurfacing can disturb accumulated infill, cut or roll turf, grind track coatings, scrape adhesive residues, and generate loading or transport losses. These are plausible release opportunities; component-resolved sports-specific emission factors are still needed. The analysis must include installation offcuts, collected solids, recovered components, and final disposal alongside the use phase.

Following their release, ENMs and MNPs are transported through atmospheric and hydrological pathways. Atmospheric transport depends strongly on aerodynamic diameter and agglomeration state. Fragments containing nanofillers may remain in the respirable fraction and be resuspended by wind or athletic activity, but respirability depends on aerodynamic size, shape, density, and agglomeration rather than the constituent filler size alone. Fu, Wang, Kumar and Cao [49] reviewed abrasion and resuspension pathways for airborne MPs around synthetic surfaces. Particles in the PM_10_ and PM_2.5_, aerodynamic fractions are a possible exposure pathway for spectators and nearby communities, but composition-resolved airborne concentrations and source attribution must be established before that exposure is quantified. Indoor sports facilities may experience continuous particulate generation; however, comprehensive breathing-zone exposure profiles for athletes remain unavailable.

Hydrological transport through surface runoff, wastewater effluent, and snowmelt is a predominant pathway for particles to enter soils and aquatic ecosystems [27,36,52]. Rainfall directly connects sports surfaces to receiving waters. Hua, Lundqvist, Naidu, Ekvall and Cedervall [48] detected EPDM-related material in synthetic-field drainage. Micro- and nanoscale particles may accumulate in drainage sumps or enter wastewater treatment plants, where retention in sludge redistributes the environmental burden rather than eliminating it [26,47]. In aquatic systems, polymer fragments may also act as vectors for chemical additives and heavy metals, including Zn^2+^ and Ag^+^, potentially contributing to the “Trojan Horse effect” (combined particle–chemical exposure); however, a carrier-mediated increase in toxicity requires case-specific evidence. The processed EPDM-related drainage signature in [48] does not provide a validated polymer-specific ambient NP count. Water-phase occurrence, wastewater retention, and personal inhalation are distinct evidence boundaries.

Adjacent soils function as both particle sinks and secondary emission sources. Transport by footwear, rain splash, and wind can concentrate infill particles along field margins [26,162,163]. NP mobility is strongly influenced by aggregation, ionic strength, and biofilm formation, which determine whether particles undergo sedimentation or remain colloidally stable [164]. Field-derived NPs may therefore behave differently from pristine nanospheres evaluated under controlled laboratory conditions. Importantly, environmental management strategies must account for the displacement of exposure, because capturing particles in wastewater filters may merely transfer the contaminant burden to sewage sludge. Environmental aging fundamentally alters particle hazard profiles. Rubber infill may frequently contain heavy metals, POPs, and antioxidants [27,28,46,47,48]. Under environmental conditions, the tire antioxidant N-(1,3-dimethylbutyl)-N′-phenyl-p-phenylenediamine (6PPD) can transform into 6PPD-quinone [33,165,166]. Tire-derived 6PPD-quinone has caused acute mortality in coho salmon in mechanistic studies [166]; this adjacent chemical evidence does not establish a sports-field dose or a uniform toxicity ranking across species. However, Nishi et al. [167] found that concentrations of regulated polycyclic aromatic hydrocarbons (PAHs) in Japanese rubber granules were below EU limits (limit of quantification (LOQ) = 0.025 μg g^−1^). This finding demonstrates that generalized hazards attributed to broad material classes cannot substitute for sample-specific compositional analyses.

Researchers have investigated nano-enabled sensors and photocatalytic nanocomposites for detecting or treating chemicals such as diclofenac [168,169,170]. These analytical and remediation examples do not establish that the detected analytes originated at a sports venue; they are included as contextual technologies for chemically resolved monitoring. Concurrently, recycling end-of-life sports equipment, such as CFRPs, into cement composites can promote material circularity [3,8,53,84,96,129]. Recycled CFRP contents of 0.25–1% have improved mechanical impact properties, whereas 0.2–0.8% have enhanced electrical conductivity [53]. Nevertheless, mechanical shredding during recycling can generate fine fibers and dust; composition-resolved analysis is needed to determine whether they contain liberated nanofillers. Material recovery does not itself demonstrate low emissions or a negligible receptor dose.

Aging may also increase the capacity of sports-derived particles to transport external contaminants. Zhang, Jiao, Wang, Gu, Zhang, Liu, Zhang and Tan [41] reported maximum Langmuir Pb adsorption capacities of 0.13, 1.50, and 2.67 mg g^−1^ for pristine SBR, aged-SBR, and EPDM, respectively. These Langmuir capacities are batch-test maxima, not field Pb concentrations or direct ecological effects. Their within-test ratios are approximately 11.5 for aged-SBR versus pristine SBR and 20.5 for EPDM versus pristine SBR; formulation and receiving-medium differences preclude a universal risk ranking. Aging increased surface cracking in SBR, thereby generating additional sorption sites. Combined with evidence that plastics can act as vectors for organic pollutants [171], all of these findings indicate that risk assessments must simultaneously consider particle-bound and freely dissolved chemicals. Agglomeration, dissolution, and eco-corona formation further modify particle mobility and ecotoxicity [36]. Future assessments should therefore prioritize field-aged, matrix-associated particles rather than pristine nanopowders.

## 4. Exposure to ENMs and MNPs: Implications for Athlete Health, Ecosystem Safety, and Sustainability

**Figure 4:**
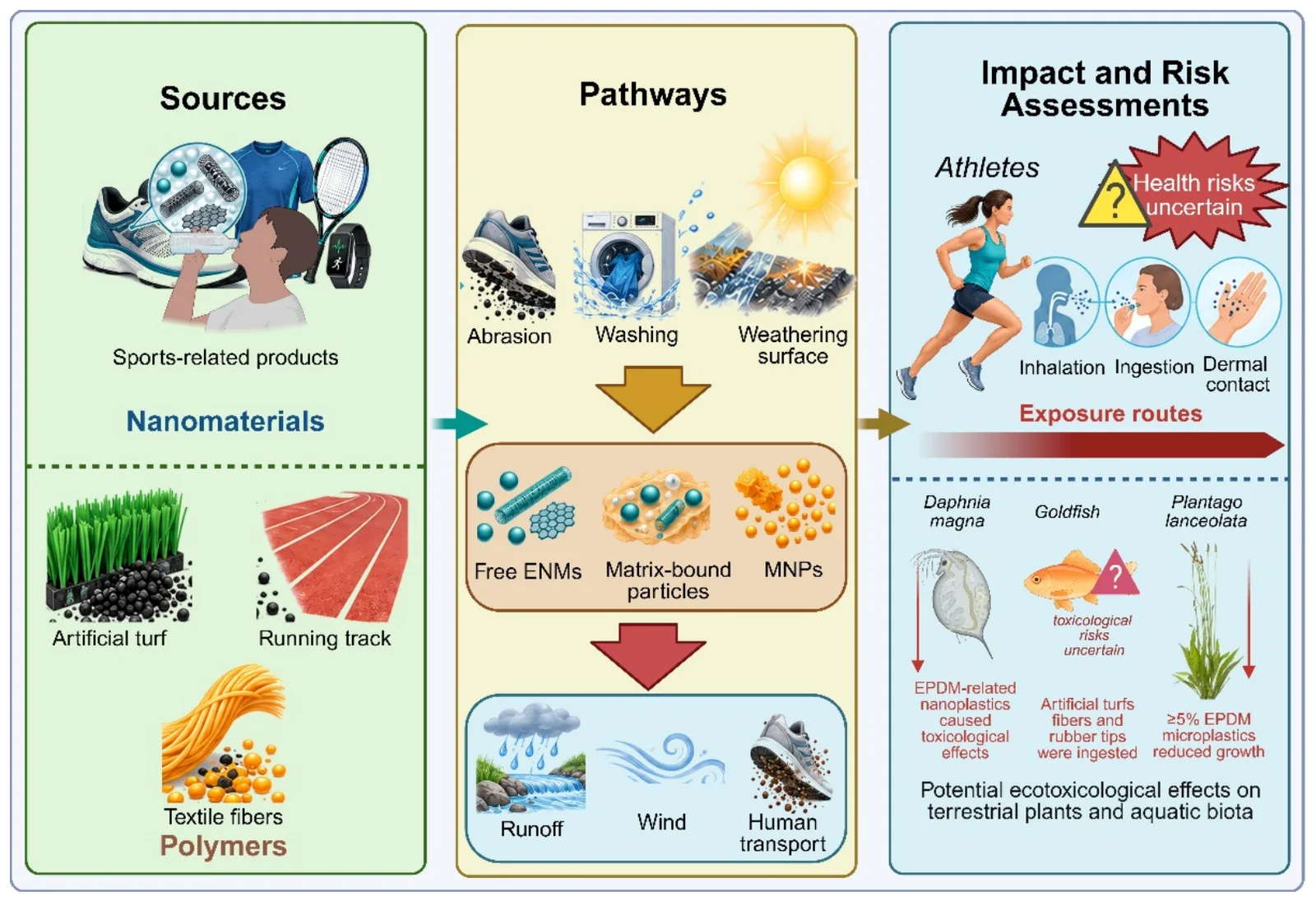
Sources, pathways, and risk assessments of nanomaterials and polymer-based materials in the sports industry.

Integrating NMs and polymers into sports equipment and facilities creates a complex benefit–risk paradigm with activity-specific exposure patterns (Figure 4). Emerging evidence suggests that NMs may affect multiple organs and tissues [1], with nanotoxicological studies linking these effects to particle size, chemical composition, and interactions across biological systems [39]. Inhalation is a possible route during exercise on dusty surfaces which principally concern production or machining [37,94], so they cannot establish the relative importance of inhalation during sports participation. Ingestion may occur through hand-to-mouth contact or accidental ingestion of infill, whereas skin contact may occur with sweat-retained particles, while abraded skin represents a separate exposure condition. Goalkeepers and facility workers exhibit distinct contact profiles compared with spectators [33,167], indicating that generic “field-user” exposure factors are inadequate.

Nano-enabled equipment can improve mechanical strength and impact resistance, suggesting potential injury-prevention benefits that require clinical validation [5,13,14,89]. Smart materials and flexible sensors facilitate real-time biomechanical monitoring by detecting mechanical overload and quantifying impact forces [15,16,57,67,134,135]. Advanced systems can also harvest energy and classify movement patterns [17,121,122,172,173]. For example, a modular resistive system measured forces up to 330 N over an area of 2 m^2^ [172], while a self-powered pressure sensor remained stable after 10,000 cycles [173]. The resistive force-mat example [172] establishes sensing performance, not an ENM identity: an Arduino Nano controller or flexible sensing architecture is not evidence of an engineered nanoparticle constituent or emitted aerosol. However, establishing clinical utility requires rigorous evaluation of device durability and nanoparticle release under realistic conditions involving sweat, thermal stress, and abrasion.

Nano-enabled wearables can assess internal physiological states through biochemical sensing [118,121,174,175]. Electronic textiles and printed sensors increasingly integrate NMs, functional coatings, and machine-learning algorithms to provide real-time feedback [159,176,177]. Nano-textiles represent a potential route of ENM exposure because release into sweat and wash water makes dermal and environmental contact plausible. However, skin penetration depends on particle size, coating, and skin integrity [116]. Metabolic sensors can analyze sweat and saliva. For example, nano-Al_2_O_3_/lactate oxidase electrodes containing 80–90 nm particles exhibited a detection limit of 0.08 mM and a 96% recovery in the tested ex vivo sweat analysis, with comparison to high-performance liquid chromatography (HPLC) [18]. Laser-induced graphene saliva strips achieved a lactate sensitivity of 33.3 ± 0.9 μA mM^−1^ cm^−2^ [19], while an optical microfiber blood-pressure sensor responded within 35–46 ms and achieved a correlation coefficient of 0.96 for systolic predictions [20]. Furthermore, terahertz (THz) nanobiosensors and machine-learning-integrated electronic textiles support advanced motion tracking [119,122,134,135,159,174,176,177]. Despite promising analytical performance, these technologies have not translated into routine sports analytics because validation across diverse physiological conditions remains insufficient. Crucially, material degradation and nanoparticle leaching during repeated use remain largely unquantified, limiting comprehensive safety evaluations. Neither sensor detection limits nor physiological-signal accuracy quantify incidental particle dose. Relevant release tests must determine whether the active layer remains bound, migrates as a fragment or particle, or releases dissolved constituents.

Incidental exposure to ENMs and MNPs may occur through inhalation, dermal contact, and ingestion. Hazard and risk must remain conceptually distinct. MNP toxicology supports plausible adverse mechanisms, including oxidative stress and epithelial barrier disruption. However, much of the available evidence is derived from pristine spherical plastics tested at high concentrations, which do not adequately represent weathered field mixtures [28,29]. These findings should not be generalized to ENMs of different composition or to all NP size fractions. Artificial-turf components may contain carcinogenic or endocrine-disrupting chemicals, yet epidemiological evidence among turf users remains insufficient to establish either categorical harm or safety [33].

Inhalation exposure is documented during composite production and machining [37,94]; its relative contribution during sports participation remains uncertain. Exercise increases inhaled air volume; breathing route and particle-size distribution can alter deposition, but measured airborne concentrations and appropriate activity records are needed to estimate inhaled and deposited particle doses. Ferreira, Homem, Cereceda-Balic, Fadic, Alves and Ratola [132] measured total volatile methylsiloxane (VMSs) concentrations of 1.60–5089 ng g^−1^ across 135 crumb-rubber and 12 alternative-infill samples and found that modeled oral and dermal exposures were low. However, the study did not evaluate inhalation exposure, NP toxicity, or mixture effects. Ye, Liang, Zhou, Li, Ma, Liu, Luo, Chen, Zeng and Li [42] modeled inhalation doses of 7–11 and hand-to-mouth doses of 18–28 particles kg^−1^ d^−1^ for children under assumed daily sports conditions. The study compared these outputs with reported HC5 and HC10 values of 15–20 and 45–60 particles kg^−1^ d^−1^, respectively [42]. These comparisons are not validated human or athlete safety thresholds; the applicability of these toxicological endpoints and dose metrics to human polymer-particle exposure has not been established. Nevertheless, these estimates were derived from a limited number of wet-season runoff samples; therefore, they should be regarded as prioritization tools rather than definitive safety thresholds. In particular, deriving an airborne source term from runoff with a resuspension assumption requires external validation; a water concentration cannot be inserted directly into an inhalation-dose calculation.

Dermal exposure may arise from NMs incorporated into sunscreens, apparel, and cosmetics [44]. Released TiO_2_, ZnO, or Ag-containing material may contact skin; sweat can change retention, surface chemistry, and speciation [36,44]. Contact alone does not establish transdermal particle penetration or systemic uptake, and exercise-induced vasodilation is not evidence of enhanced permeation. No direct athlete-specific evidence cited here demonstrates exercise-enhanced ENM penetration through intact skin. Oral exposure can occur through hand-to-mouth transfer and mucociliary clearance. Although nanosensors can effectively detect contaminants in sports supplements, such as nano-gold sensors for vitamin C with a detection limit of 1.86 μg mL^−1^ [133] and magnetic nanoparticles for aflatoxin M1 with a limit of detection (LOD) of 0.002 μg kg^−1^ [141], these analytical applications do not establish consumables as major exposure sources. Hazard assessments must ultimately account for the bioavailable dose and physicochemical transformations associated with athletic physiology. Children require separate evaluation because of their higher relative ventilation rates and more frequent hand-to-mouth behavior [49]. Elite athletes inhale greater volumes of air during intense exercise; the amount of particle inhaled depends on airborne concentration, and remains unquantified for source-attributed sports-derived MNPs. Moreover, no robust biomonitoring studies have linked CNTs, graphene, or metal oxides released from sports equipment to internal doses in athletes [9,10,11]. Actual exposure levels must be separated from source quantities. Known observations include chemically identified MP counts in selected surface runoff [41,42], product release under laboratory conditions [36,116], and production-zone aerosol measurements [37]. Unknown quantities include the source-attributed airborne concentration during play, athlete inhaled and deposited NP/ENM dose, hand-to-mouth transfer, dermal permeation, and cumulative internal burden. An inhaled amount can be estimated only from a compatible air concentration and inhaled air volume over activity duration; a deposited amount additionally requires size- and breathing-dependent deposition. Comparable baselines should be sampled simultaneously on nearby natural or non-sports surfaces and in upwind, indoor, or urban background air, with other consumer and traffic sources measured separately. Runoff particles L^−1^, field losses kg year^−1^, and laboratory-prepared particles mL^−1^ cannot be converted directly into an athlete dose.

Intentional nano-bio interfaces in sports medicine require distinct benefit–risk evaluations. Nanocomposite scaffolds, including reduced GO/nano-hydroxyapatite (RGO/nHA) and silk-fibroin/polylactic acid-polycaprolactone (PLA-PCL) nanofibers, have shown properties relevant to tissue repair in experimental studies [21,22,23,24,145]. A 3D-printed PLA scaffold containing approximately 80 nm HA particles achieved a strength of 6.0 MPa at 15 wt% and increased mesenchymal-cell growth by 25% [23]. Graphene-based nanocomposites may also provide alternatives to conventional grafts and metallic screws [145], while NM-based interventions were reported to increase the study-defined meniscal-repair effectiveness by 8.22% [144], an endpoint that should not be interpreted as a universal clinical effect. Tendon-mimetic nanowoven scaffolds have promoted M_2_ macrophage polarization [24], and nanobiotic strategies have been proposed for managing tendon injuries in Taekwondo athletes [149]. Advanced bone–implant interfaces, such as Mg-Zn scaffolds coated with nHA/chitosan, reduced the laboratory corrosion rate from 2.09 to 0.58 mm year^−1^ [99]. Bioactive-glass delivery systems, modified titanium surfaces, and injectable calcium-phosphate systems [25,160,161] concern other formulations and endpoints and should not be assigned to this Mg-alloy result. Nevertheless, short-term biocompatibility evidence is insufficient to establish long-term safety. Important knowledge gaps remain regarding in vivo degradation kinetics and the potential migration of unbound nanoparticles. Muscle-regeneration nanofibrous scaffolds and bioprinting strategies [150,151] extend the therapeutic context. Their intentional administration, degradation products, local chemistry, and clearance require application-specific assessment; their efficacy does not imply diffuse environmental exposure in sports.

Applications also extend to neuronal repair, anti-doping surveillance, and dental materials. Nanoscale extracellular vesicles are being investigated for neuronal repair [178]; these biological vesicles should not be conflated with the muscle-regeneration scaffolds in [150,151] or with freely released inorganic ENMs. Nanocomposite sensors, including AgNP, surface-enhanced Raman spectroscopy (SERS) chips, and copper oxide (CuO)-graphene platforms, can detect prohibited substances such as clenbuterol, ractopamine, and salbutamol at low concentrations (mg L^−1^), with performance advantages reported under specific analytical conditions [139,140,143,179,180,181]. A meta-analysis reported higher analytical performance for the nanocomposite sensors than for the conventional doping sensors included in its selected comparison (Z = 4.14, *p* < 0.0001) [143]; this statistical result concerns the included assays, rather than nanoparticle exposure or universal superiority across anti-doping methods. Moreover, a review of microscale separation and mass-spectrometric workflows reports sample and solvent savings > 90% and sub-pg mL^−1^ detection limits for selected steroids, selective androgen receptor modulators, and peptide assays [181]. Nano-electrospray describes an ionization configuration, not necessarily a nanomaterial-containing product or an ENM emission source. In addition, acid challenge, aging, and simulated bruxism affect restoration properties in the tested dental composites [182,183]. Oral particle release remains a hypothesis requiring direct identification and dose measurement; restoration fatigue or imaging does not establish a sports-specific swallowed ENM dose. The relevance of these analytical and dental applications to source-to-exposure assessment therefore lies in device integrity, laboratory waste, or directly measured oral debris, rather than in their analytical sensitivity or therapeutic purpose alone.

Critical evaluation of the available evidence reveals a pronounced imbalance. Although functional performance has been extensively quantified [18,19,20,119,121,129,173,175], incidental exposure and internal dosimetry remain underexplored. Athletes cannot be treated as standard consumers because activity, ventilation, skin condition, and contact behavior can alter exposure dynamics in material- and route-dependent ways. Establishing a rigorous benefit–risk framework requires paired study designs that simultaneously evaluate device functionality, nanoparticle release under sport-specific stressors, and personal dosimetry. Direct-exposure studies should integrate personal inhalation samplers, hand wipes, and targeted dermal measurements, with participants stratified by sport, playing position, and environmental conditions. Background and other consumer sources, including MNPs contamination in bottled water and protein supplements [184,185], require separate attribution; bottled-water exposure is not an established dose baseline for sports participation. An integrated analytical and probabilistic risk assessment of commercial sports protein supplements in China reveals ubiquitous co-exposure to toxic metals and MPs [185], underscoring elevated health risks under high-consumption scenarios that necessitate stringent contaminant mitigation alongside scenario-driven regulatory oversight.

Ecotoxicological studies identify potential hazards along the source-to-receptor pathway (Table 4 and Figure 4). Their relevance to sports venues depends on measured environmental concentrations. Terrestrial exposure is biologically relevant at localized hotspots. van Kleunen, Brumer, Gutbrod and Zhang [45] exposed *Plantago lanceolata* and other grassland species including perennial grasses (*Alopecurus pratensis* L., *Festuca guestfalica* Boenn. ex Rchb., *Lolium perenne* L.), perennial forbs (*Galium album* Mill., *Leucanthemum ircutianum*, *Prunella vulgaris* L.), and a biennial forb (*Daucus carota* L.) to EPDM (ranging 0.5–2.5 mm size and a density of 1.6 g/cm^3^) infill and found that concentrations ≥ 5% substantially reduced plant growth and survival while altering competitive interactions. Growth, survival, and competition responses depended on material and loading; these are greenhouse observations, not a venue-wide 5% soil-effect threshold. Mechanical degradation introduces polymer fragments into drainage systems, where they may affect aquatic biota [27]. Takahashi, Fujinuma, Yoshitomi, Shimizu and Kobayashi [46] and Chiba, Fujinuma, Yoshitomi, Shimizu and Kobayashi [47] demonstrated that goldfish and wild crucian carp ingest artificial-turf fibers and rubber granules under controlled laboratory conditions, although field observations of this phenomenon remain limited. Notably, laboratory-based ingestion does not inherently establish trophic transfer or population-level impacts at sports venues. No adverse effects or toxicological risks were reported in these studies, further research is required to fully evaluate the broader ecological implications. Furthermore, product-released ENMs (PR-ENMs) elicited toxicological effects in *Raphidocelis subcapitata* and *D. magna* to varying degrees under laboratory conditions, with more pronounced effects on *D. magna* than *R. subcapitata* [36]. However, these findings are limited because they exclude environmental transformation, long-term accumulation, and trophic transfer dynamics. Consequently, future research must evaluate PR-ENMs under realistic ecological conditions to accurately assess environmental risks, and integrating safety-by-design principles in nano-enabled product manufacturing is recommended to mitigate ecological exposure. Moreover, Hua, Lundqvist, Naidu, Ekvall and Cedervall [48] characterized a mechanically generated EPDM abrasion preparation with an NTA count of 4.3 × 10^8^ ± 5.8 × 10^7^ particles mL^−1^ and a mean size of 233 ± 6 nm. Outer-layer preparations and their leachate were toxic to *D. magna* under the tested conditions [48], whereas the inner-layer preparation showed no significant toxicity. These preparation values are not measured ambient drainage NP concentrations. These findings implicate surface-associated chemicals and leachates in the observed toxicity but do not identify the specific toxic agents. Bulk ATR-FTIR signatures and NTA size/count results complement each other, but neither independently confirms the polymer identity of every detected NP. Therefore, calculating an exposure margin or field ecological-risk conclusion requires matched ambient particle identity, size distribution, concentration, and chemical-control design.

Metal-containing nano-enabled product releases, including TiO_2_, ZnO, and Ag systems, can adversely affect aquatic test organisms under product-specific laboratory conditions [36]. Particle and dissolved chemical contributions must be separated. Zhang and Tan [40] reported runoff characterization, ecological screening, and batch-test metal sorption. Screening classifications and adsorption capacities do not directly demonstrate ecological injury in receiving waters. Ye, Liang, Zhou, Li, Ma, Liu, Luo, Chen, Zeng and Li [42] used a predicted no-effect concentration of 1.9 × 10^5^ particles·m^−3^ (equivalent to 190 particles L^−1^). Dividing the reported mean runoff counts of 734 and 652 particles L^−1^ by 190 gives screening risk quotients (RQs) of approximately 3.86 for lawns and 3.43 for tracks. The denominator’s endpoint relevance and uncertainty must be retained: these water-phase MP screening ratios do not establish human risk, ENM risk, or effects downstream after dilution and settling. In aquatic environments, organisms are simultaneously exposed to polymer fragments, dissolved ions, and surface residues. The environmental release of antimicrobial NMs, such as Ag, raises material- and organism-specific ecological questions after discharge [186]; tested antimicrobial activity does not establish ambient mixture toxicity. Turf-derived particles can also accumulate in soils, motivating tests of, microbial communities, invertebrates, and organic-matter dynamics during repeated wetting cycles [27]. Those endpoints require measured field gradients and appropriate controls.

Distinguishing hazard from risk is paramount when evaluating ENMs and MNPs, especially because ecotoxicological models often overestimate risk through supra-environmental dosing. Robust risk assessment requires integrating hazard screening with environmentally relevant exposure modeling and realistic dosimetry. In sports environments, while localized ENM and MNP sources, such as airborne particulates, turf runoff, and supplements, are identifiable, environmental detection does not equate to biologically relevant internal exposure. Significant uncertainties remain about MP absorption, distribution, metabolism, and elimination, driven by reliance on non-clinical models, methodological heterogeneity, and the undefined physiological relevance of gastrointestinal translocation. Addressing these gaps requires harmonized analytical methodologies, validated biomarkers, and quantified MP kinetics. This is especially critical for athletes, who experience a unique confluence of repeated dietary MP intake, exercise-induced intestinal permeability, and elevated metabolic demands. Future sports-specific research must therefore integrate comprehensive exposure assessments with evaluations of gut barrier integrity, systemic inflammation, and clinical recovery. Until environmental exposures are rigorously aligned with health-based thresholds and internal doses are definitively quantified, risk assessments for athletic populations must remain provisional.

The end-of-life management of nano-enabled products and polymeric sports materials presents a “circularity paradox.” For instance, downcycling end-of-life tires into SBR granules diverts waste from conventional disposal pathways but transforms a concentrated material stock into diffuse environmental emissions [33,35,51]. Consequently, evaluating circularity solely by mass recycled may inadvertently reward converting visible waste into less visible MP emissions. This paradox extends across diverse material classes. Although nanocomposites can improve durability and reduce upstream resource demand [2,103], their persistence may increase environmental longevity following fragmentation. Biobased resins and natural fibers, including nanocellulose and piassava, reduce dependence on fossil resources [3,8,79,80,84,85] but often retain persistent synthetic matrices or nanofillers, thereby limiting biodegradability. Furthermore, the mechanical recycling of CFRPs or thermoplastics can generate inhalable particulate matter [37,53], while recycled polyethylene terephthalate (PET) textiles provide a parallel setting for microfiber release [50]. In these cases, recycled content alone does not determine the magnitude or trajectory of emissions compared with virgin counterparts.

In sports applications involving ENMs and plastics, circular transitions do not eliminate environmental burdens; rather, they shift particulate emissions to secondary processing and end-of-life stages. The 2021 material flow analysis of the Norwegian artificial turf system reveals that while the majority of SBR granules are retained or managed through recycling (57%), winter maintenance recovery (8%), incineration (4%), and private repurposing (3%), a substantial 19% (1187 t) is directly lost to the environment via runoff, deposition, and equipment transfer [51]. Operations such as sorting, shredding, and artificial turf demolition act as point sources of particulates and MPs through material attrition and harmful dust generation. Terminal processes, such as incineration, produce airborne particulates and contaminated ash. Additionally, the altered physicochemical and mechanical properties of recycled materials may defer emissions to subsequent use and wear cycles. Ultimately, circular systems often exchange the concentrated upstream impacts of primary production for dispersed downstream particulate leakage. Effective circularity assessments must therefore employ comprehensive life-cycle assessments to quantify particulate releases across processing, use, maintenance, and end-of-life stages, alongside stringent containment measures. This ensures environmental burdens are genuinely reduced rather than merely displaced. The circularity paradox thus represents a conditional trade-off requiring rigorous comparative analysis, acknowledging that both virgin and recycled formulations can generate particulate emissions and chemical leachates.

## 5. Integrated Risk and Policy Governance Strategies and the Circularity Paradox

A fair comparison of virgin and recycled polymers uses an equal sports service, such as one square meter of compliant playing surface over a specified lifetime and activity intensity. Virgin material requires extraction, production, and compounding; recycled material adds collection, sorting, cleaning, shredding, and reprocessing, which can also generate dust and residues. Both routes incur losses from installation, play, maintenance, and removal. Recycled tire-to-infill use can reduce demand for virgin feedstock while moving a concentrated stock into a dispersive application; a well-bound recycled component may behave differently. No general higher- or lower-emission ranking is assigned without matched formulations, aging, service life, and measurements [2,31,33,35,37,50,51,53,66].

For each route, a material balance should record purchased material, remaining installed stock, captured residues, unrecovered off-site losses, recovered components, and final disposal. Fragmentation converts existing particle mass into smaller sizes and should not be double-counted as additional mass loss; particle number can increase while mass is conserved. Stage-specific diffuse particle losses should be summed within the same lifecycle boundary, with dissolved chemical release recorded separately. Demolition or resurfacing can produce concentrated disturbance events through infill extraction, turf cutting, adhesive removal, track grinding, loading, transport, and shredding. Reuse or recycling should be accompanied by contained extraction, covered storage and transport, dust collection, component segregation, and verified recovery destinations. Their efficiencies need measurement rather than a generic recyclability claim [2,35,37,53,66].

**Figure 5:**
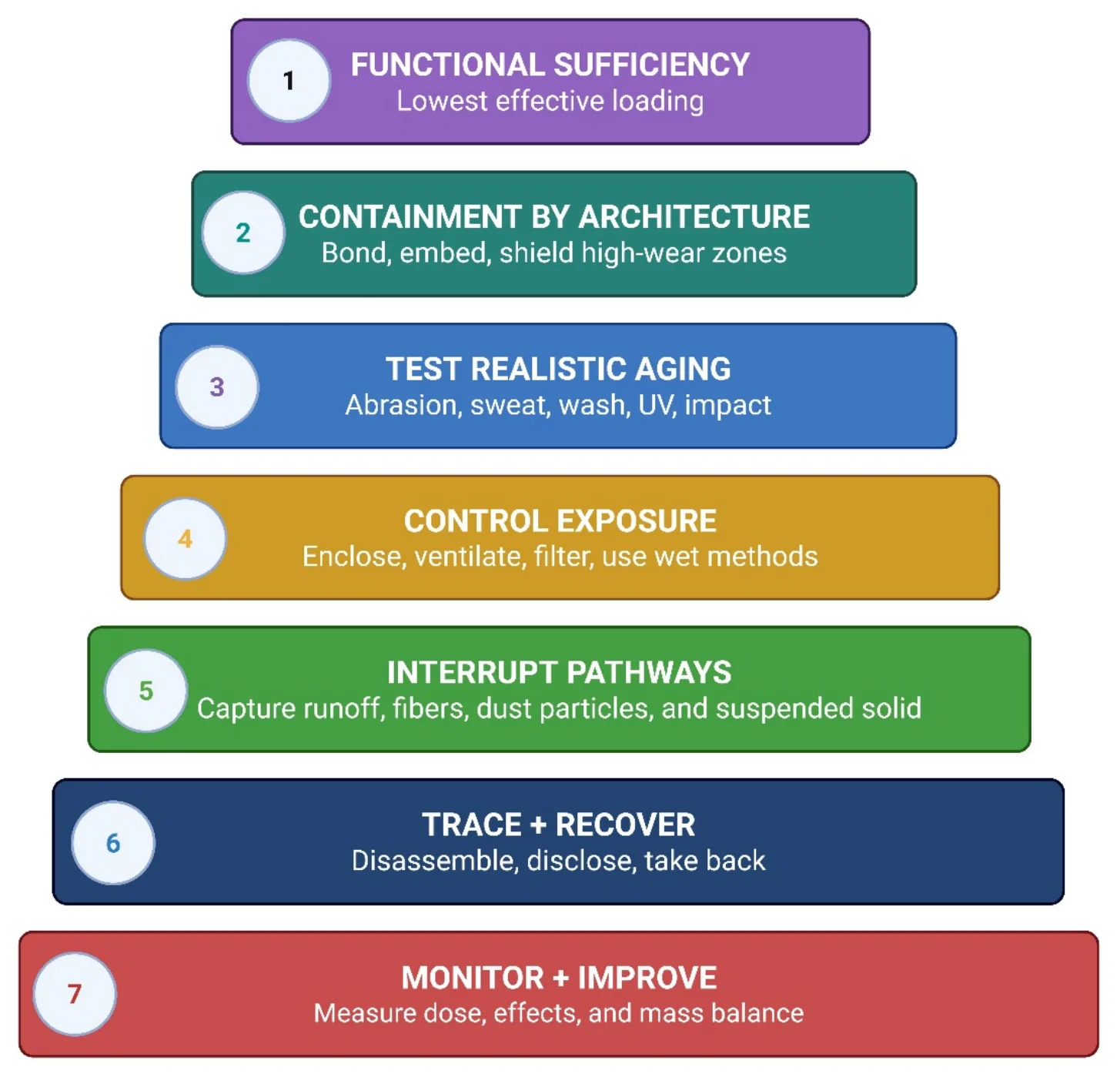
Release-first governance hierarchy.

A “release-first” governance hierarchy should prioritize functional necessity, minimize hazardous content, and strengthen containment (Figure 5). Functional sufficiency should be achieved using the lowest required NM loading, as benefits can be attained at formulation-specific concentrations, including the reported 0.5% nano-SiO_2_ footwear example and the distinct laminate formulations [5,79,89]. A useful loading in one test is not a universal low-emission optimum. Prevention should precede capture. Low-shedding fibers, strongly bound nano-coatings, and safer-by-design formulations should be evaluated under sport-specific conditions before associated sustainability claims are accepted [27,49]. Substituting synthetic materials with cork, coir, or sand may reduce the release of persistent synthetic MPs. However, procurement assessments should comprehensively compare particle release, chemical composition, thermal behavior, and end-of-life recovery across material alternatives. Matched comparisons should also document retained function over service life: early replacement can offset apparent resource savings, while a durable matrix may still emit small fragments.

Where synthetic systems remain necessary, effective containment is essential. Edge barriers, entrance grates, drain filters, and controlled snow storage can reduce off-site particle transport [27,35,47]. These measures require standardized assessments of capture efficiency and documented maintenance protocols. Behavioral interventions alone cannot replace engineering controls for drain management and infill replenishment [32,65,86,90]. Although wastewater treatment retains larger particles, transferring them to sludge does not eliminate them. Consequently, the environmental mass balance must incorporate influent, effluent, sludge, and final disposal pathways. Coarse-infill capture must be evaluated separately from fine-fragment retention, with size- and mass-resolved efficiency, bypass, snow handling, maintenance, and safe management of collected residues.

Realistic release testing must reproduce relevant aging processes and conditions of use. Footwear should undergo cyclic gait abrasion; textiles should undergo repeated washing and UV exposure; and turf systems should undergo simulated play. Released materials should be characterized according to particle number, mass, size, polymer and nanofiller identity, free versus matrix-bound ENM content, and dissolved-ion fraction. Occupational exposure during production and maintenance should be controlled using enclosures and local exhaust systems, supported by near-field and far-field monitoring models [37].

Policy frameworks should regulate lifecycle emissions rather than isolated ingredients. Zuccaro et al. [187] provide historical evidence of international variation in artificial-turf, PAH, and per- and polyfluoroalkyl substances (PFASs). Restrictions on intentionally added polymer MPs do not directly resolve all secondary fragments from fibers and tracks [35]; the EU measure should not be described as a general ban on artificial-turf use, all secondary debris, or every nanoform. Multi-objective design strategies should evaluate functional gains, release rates, and end-of-life recovery simultaneously. Computational tools used to optimize stadium panels and footwear soles [14,103,105,108,109,110] could incorporate empirically measured release data. Procurement frameworks should integrate composition limits, standardized abrasion testing, drainage-capture performance, and extended producer responsibility. Manufacturers should also transparently disclose polymer compositions and nanoform identities [125]. Remaining governance needs include entity-specific release reporting, nanoform and ingredient disclosure, lifecycle recovery records, and evidence linking measured emissions to receptor dose. These are proposed priorities; a binding universal sports-specific nano-emission limit is not asserted.

Analytical harmonization should be a primary research priority [34]. Released materials must be reported according to particle number, mass, size, and polymer identity, with separate investigation of free ENMs, ENMs retained in polymer fragments, MNPs, and dissolved chemicals. Sport-specific protocols should specify the sampled area, intensity of use, field age, and minimum detectable particle size. Reference materials should include aged SBR and EPDM rather than relying solely on pristine polystyrene. A tiered analytical framework should combine microscopy with µ-FTIR or Raman identification of suitable MP fractions, thermal methods for polymer mass, and electron microscopy with compositional analysis or validated particle-resolved methods for nanoforms. NTA or dynamic light scattering supplies size information but does not independently prove polymer identity. Matched background surfaces, air, and runoff should also be monitored to distinguish venue-related emissions from the urban background. The minimum framework established MP reporting principles to sports materials and their different release forms [188], keeping analytical uncertainty separate from biological uncertainty.

A minimal study should report the actual sampled and identified size window. Proposed reporting bins are <100 nm, 100 nm to <1 µm, 1 to <20 µm, 20 to <100 µm, 100 to <500 µm, and 500 µm to <5 mm, with ≥5 mm material recorded separately for inventory closure. These bins are proposed here to improve comparison, not as a claim that one method can recover and chemically identify every fraction. Established reporting principles [188] support transparent blanks, recovery, detection limits, and chemical confirmation; unrecovered fractions remain unmeasured rather than being reported as absent. Analytical uncertainty concerns what was collected and identified: upstream sieves and downstream filters, size-dependent recovery, digestion damage, blank contamination, spectral thresholds, chemical-confirmation fractions, and elemental or polymer specificity. A 0.45 µm filter cannot establish sub-48 µm recovery after an upstream 48 µm sieve. Thermal analysis can provide polymer mass without preserving count or morphology; NTA can count detectable particles without proving each particle is polymer. These measurement limitations should be reported separately from biological interpretation [30,34,42,124,188]. Biological uncertainty concerns delivered dose, exposure duration and route, field-aged composition, particle-versus-chemical causation, species or human susceptibility, clearance, and chronic outcomes. A precise environmental count does not resolve these questions if the hazard experiment uses a different distribution, surface chemistry, or medium. Testing should include environmentally measured low-dose ranges and relevant hotspots, field-aged particles and leachates, washed-particle and dissolved-chemical controls, and verification of delivered rather than nominal dose. A high-dose–response does not establish routine field harm; a negative result at one endpoint does not establish broad safety [28,29,33,39,45,46,47,48].

Governance and research frameworks must close the environmental mass-balance loop. Field-based ecological studies should quantify particle gradients extending from turf edges and evaluate their effects on soil microbiomes and plant communities. Aquatic studies should link drainage emissions to sediment accumulation and trophic transfer [45,46,47]. Material-flow analyses must be validated against field-monitoring data, while LCAs should explicitly account for MP leakage and use clearly defined allocation rules [31,51]. Circularity indicators should account for diffuse particle loss and hazardous chemical content so that dispersive downcycling is not credited solely by recovered mass. Finally, geographic coverage must extend beyond Europe and Asia to capture the effects of diverse climatic conditions on UV aging and hydrological transport [35,49]. Research priorities are source-attributed field emission baselines, activity-resolved personal monitoring, and chronic testing matched to measured aged-particle and leachate distributions. Product designers should prioritize binding integrity and verified abrasion resistance; operators should document top-ups, captured residues, bypass, and removal losses; and regulators should distinguish intentional-particle restrictions from the remaining secondary-particle and lifecycle reporting gaps.

## 6. Research Gaps and Concluding Remarks

ENMs offer functional benefits for sports equipment and sensing, while many tissue-repair applications remain under investigation. Their environmental release depends on material composition, matrix binding, aging, and use conditions. Mechanical abrasion, ultraviolet aging, and laundering can generate matrix-bound fragments or MNPs and, under tested conditions for some materials, release free ENMs or dissolved constituents. Each emitted form requires direct identification. Field studies of artificial turf primarily document polymer-particle emissions; these observations do not establish the magnitude of free ENM release. Distinguishing ENMs from NPs and larger MPs requires size- and composition-resolved measurements. Reported environmental concentrations also vary by several orders of magnitude, reflecting differences in study settings, sampling strategies, and analytical methods. Environmental weathering further governs particle fragmentation, aggregation, and contaminant sorption, making the physicochemical properties of released particles as critical as the original materials’ composition.

A sustainable framework for sports nanotechnology must integrate material performance, human health, and environmental safety. Athletes may be exposed through inhalation, ingestion, and dermal contact. Although mechanistic toxicological evidence warrants continued investigation, current data are insufficient to establish a generalized causal disease burden, as personal dosimetry and longitudinal epidemiological studies are scarce. Furthermore, chemical-specific risk assessments cannot adequately characterize nanoparticle exposure under the high-ventilation conditions associated with exercise or the chronic effects of complex particle–chemical mixtures.

Ecotoxicological evidence identifies potential hazards but remains fragmented. Experimental studies under controlled conditions report impaired plant growth at high EPDM burdens and toxicity of mechanically generated EPDM-NPs and surface leachates to freshwater invertebrates. These findings cannot be generalized to all ENMs or to typical sports-venue exposures. Substantial uncertainties remain regarding environmentally realistic exposure concentrations, trophic transfer, and the combined toxicity of micro- and nanoscale particles and sorbed contaminants. Transformation processes require particular attention because environmentally aged particles can differ substantially from pristine materials in their bioavailability and toxicity. In particular, the EPDM abrasion-preparation count must not be used as the ambient drainage concentration, and organism responses in that preparation cannot establish ecological effects at measured field concentrations. Additionally, although isolated oral and dermal exposure to VMSs in crumb rubber remains below established The European Chemicals Agency (ECHA) safety thresholds, the potential synergistic “cocktail effect” with co-occurring microcontaminants represents a critical toxicological knowledge gap. Comprehensive evaluations of these combined chemical risks are therefore essential to ensure the long-term safety and sustainability of synthetic turf applications.

These knowledge gaps support prevention-oriented strategies. Key priorities include designing low-release materials, improving containment at sports venues, standardizing environmentally realistic release tests, and developing technically viable systems for end-of-life recovery. The “circularity paradox” remains unresolved: recycling waste tires reduces virgin resource use but may increase diffuse particulate emissions when the recycled material is used in dispersive applications. Circularity should therefore be evaluated not only by waste diversion but also by particle release, chemical emissions, and environmental persistence. A virgin-versus-recycled ranking requires the same functional service, system boundary, lifetime, and emission measurements, not just recycled-content percentages.

The next generation of sports materials must satisfy four interconnected requirements: high functional performance, minimal particulate and chemical release, low risks to athletes and ecosystems, and technically feasible end-of-life recovery. Meeting these objectives requires closing critical research gaps, including developing harmonized analytical methods, conducting real-world field measurements of ENMs and MNPs, performing athlete-specific dosimetry studies, and conducting pollution-aware lifecycle assessments. Integrating these priorities with safe-and-sustainable-by-design principles is essential to realizing the technological benefits of sports nanotechnology without imposing disproportionate health and environmental costs on athletes, communities, and ecosystems. The resulting source-to-receptor framework preserves the benefits and application diversity of sports nanotechnology while specifying which measurements, mechanisms, and risk inferences are actually supported.

The key messages are as follows: ENM functionality, polymer fragmentation, and dissolved chemical release are separate questions. Field evidence supports sports-surface MP occurrence and runoff transport, while ambient NP identity, freely released ENMs, and athlete internal dose remain less resolved. Laboratory hazards and modeled doses retain their study labels. Primary granule displacement and secondary generation require separate measurement and controls. Circularity requires balancing resource use, loss, and recovery across the whole lifecycle. Verified binding, abrasion resistance, containment, and recovery are actionable design principles, but quantitative human or ecological-risk conclusions still require identical exposure and response.

## Figures and Tables

**Table 1:** Comparative changes in engineered or aged material properties within study boundaries.

S. No	Material and Comparison	Property Under Test	Reported Change	Source and Setting	Release Implication	References
1.	EVA sole with nano-SiO_2_ versus base formulation	Mechanical reinforcement	0.5% loading reported to improve tensile performance; no cross-product optimum inferred.	Sports-footwear laboratory study	Free-SiO_2_ release not established.	[5]
2.	Aramid sandwich with TiB_2_ versus unmodified comparator	Flexural strength, stiffness, impact toughness	Reported increases 27.6%, 62.5%, and 104%, respectively.	Composite test study	Strength gain is not a release reduction.	[89]
3.	Natural-fiber epoxy with different graphene loadings	Mechanical properties and water uptake	Best reported tensile/flexural behavior at 6 wt%; greatest hardness and least water absorption at 8 wt%.	Laboratory composite study	Different endpoints have different optima.	[86]
4.	Bio-based polyurethane foam with functionalized MOF versus comparator	Flammability and smoke production	At 25% UiO-66-NH_2_@DOPO addition: LOI 31.2%; total smoke production reduced by 80.64%.	Laboratory foam test	Fire-test result; no sports-field emission measurement.	[68]
5.	SBR versus aged SBR	Maximum Langmuir Pb sorption	0.13 to 1.50 mg g^−1^ (~11.5-fold), under the reported batch conditions.	Sports-surface material adsorption	Not field Pb burden or toxicity.	[41]
6.	SBR versus EPDM	Maximum Langmuir Pb sorption	0.13 versus 2.67 mg g^−1^ (~20.5-fold); different formulations.	Batch adsorption	No universal material risk ranking.	[41]
7.	Outer versus inner EPDM abrasion preparations	*Daphnia* response	Outer preparation and leachate toxic under tested conditions; inner preparation showed no significant toxicity.	Mechanical breakdown experiment	Chemistry and preparation matter; no ambient-risk conclusion.	[48]
8.	EPDM versus cork granules in substrate	Plant growth and survival	Effects differ with material and volumetric loading; EPDM responses become adverse at higher loadings.	Greenhouse study	Not a venue-wide soil threshold.	[45]
9.	3D-printed PLA/nHA scaffold versus control	Compressive strength and cell proliferation	At 15 wt% HA: strength 6.0 MPa; mesenchymal-cell proliferation +25% at 42 h.	In vitro scaffold study	Intentional therapeutic boundary; not field exposure.	[23]
10.	Mg-Zn scaffold versus nHA/chitosan-coated scaffold	Laboratory corrosion rate	2.09 to 0.58 mm year^−1^ under the tested polarization conditions.	Laboratory material study	Material-specific corrosion; no athlete particle-dose inference.	[99]

**Table 3:** Release entities, measured matrices, identification methods, likely receptors, and evidence types of NMs and polymers.

Material and Release Entity	Matrix Actually Studied	Identification Boundary	Likely Receptor	Evidence Type	References
Loose SBR/EPDM/TPE granules	Turf margins, material inventories, infill samples	Source granule identity or chemical analysis; size-resolved off-site loss often incomplete.	Soil/vegetation; field users in contact.	Field observations, laboratory tests, and inventories kept separate.	[45,47,51,132]
PE turf fibers and secondary track/rubber fragments	Field runoff or ditches	FTIR methods in runoff studies; source morphology and product comparison for fibers.	Receiving-water biota; contact with deposited debris.	Field occurrence; ingestion experiments separate.	[41,42,46]
Incidental EPDM NPs and co-released chemicals	Processed drainage and mechanically generated suspension	ATR-FTIR signature plus NTA/TEM; particle count is not independently polymer-specific.	Aquatic invertebrates in laboratory; field receptor inferred.	Limited field signature plus laboratory release/hazard.	[48]
Ag, TiO_2_, ZnO from nano-enabled products	Laboratory aqueous release; textile-use/wash studies	Product microscopy/elemental or chemical methods; total metal requires dissolved/particulate separation.	Skin contact and wastewater biota are proposed venue pathways.	Laboratory and review evidence; not athlete dosimetry.	[36,116,125]
Nano-SiO_2_/CNT/graphene in footwear and composites	Material specimens; generic release tests	Matrix-bound and free constituents must be independently characterized.	Athlete or worker contact depends on actual emitted form.	Performance or generic release; sports-field free-ENM route unresolved.	[5,66]
Fe_3_O_4_-enabled TPU/PA12 production aerosol	Pilot production-zone air	Particle-size/number measurements establish aerosol; composition attribution needs additional evidence.	Manufacturing personnel.	Direct occupational process measurement.	[37]
Susceptible metal phases and dissolved ions	Product leachates and water tests	Operational dissolved fraction plus speciation; total element is not ionic identity.	Aquatic biota or skin; route-dependent dose.	Laboratory chemical release; distinct from particle release.	[36,125]
Biomedical scaffold constituents	Cells, tissues, or experimental devices	Application-specific structure, degradation, or biological assay.	Patient receiving intentional treatment.	Therapeutic evidence; not diffuse sports-environment exposure.	[21,22,23,24,25,143,144,145,146,147,148,149,150,151,159,160,161]

**Table 4:** Exposure hazard and risk interpretation for human and ecological receptors with pathway confidence.

Pathway and Entity	External Exposure Evidence	Hazard Evidence	Risk Interpretation	Confidence in Pathway	References
Runoff MPs from turf/track to water	Chemically identified counts at selected venues; fibers in drainage context	Laboratory ingestion/response in other designs	Transport supported; effects at downstream concentrations unresolved	Strong for sampled-site MP occurrence; magnitude transfer limited	[41,42,45,46,47,48]
NPs from EPDM to aquatic biota	Trace drainage signature; ambient polymer-specific NP dose unresolved	Mechanical suspension/leachate toxicity	No matched ambient exposure–response ratio	Weak for quantified environmental NP exposure	[48]
Infill MPs to plants/soil	Localized accumulation reported; broad field gradients missing	Substrate experiments at specified volumetric loads	Hazard identified; venue-wide effect not demonstrated	Moderate for local contact; weak for quantitative field risk	[45,47]
Turf fibers/infill ingested by fish	Limited field occurrence and drainage context	Controlled ingestion experiments	Ingestion demonstrated under tested conditions; chronic population effect and trophic flux unresolved	Moderate for ingestion pathway; weak for quantified ecosystem effect	[46,47]
Free ENMs from textiles to wastewater	Laboratory release for some products; venue source attribution missing	Product-specific aquatic tests	Release supported under test; field concentration and mixture risk unresolved	Moderate for tested-product release; weak for sports-venue dose	[36,116]
MPs/NPs inhaled by athletes or children	Resuspension hypothesis and modeled child doses; matched personal data limited.	General route-relevant mechanisms	No validated athlete threshold or disease burden.	Weak for composition-resolved sports personal dose	[28,29,39,42,49]
Free ENMs inhaled from footwear/equipment	Sports-field particle identity and liberated fraction not established	Generic nanoform-dependent hazard	Generic hazard cannot establish sports source or dose.	Weak	[5,39,66]
Skin contact with released ENMs	Laboratory textile/product release and topical contact	Permeation depends on material and skin condition	Contact supported; exercise-enhanced systemic penetration unproven	Moderate for tested-product contact; weak for athlete uptake	[36,44,116]
MPs ingested by field users	Modeled hand-to-mouth transfer; personal absorbed dose absent.	Generic MNP toxicology	Model is screening; no validated human HC5/HC10 interpretation.	Weak for actual sports-specific dose	[28,29,42]
Production aerosol to workers	Measured process-zone aerosol	Nanoform attribution and delivered dose require composition	Workplace controls justified; athlete transfer invalid	Moderate for process aerosol exposure; nanoform attribution limited	[37]
Lifecycle shifts and ecosystem management	Runoff MPs, infill inventories, and material-flow models	Chemical, particle and biota tests have different boundaries	Sustainability requires separate mass balances and exposure indicators; recycled content alone is not a risk estimate	Moderate for recorded inventories/occurrence; weak for a general virgin/recycled emission ranking	[31,35,41,42,45,46,47,48,51]

## Data Availability

No new data were created or analyzed in this study. Data sharing is not applicable to this article.
